# Regulation of O-Linked N-Acetyl Glucosamine Transferase (OGT) through E6 Stimulation of the Ubiquitin Ligase Activity of E6AP

**DOI:** 10.3390/ijms221910286

**Published:** 2021-09-24

**Authors:** Kangli Peng, Ruochuan Liu, Caiwei Jia, Yiyang Wang, Geon H. Jeong, Li Zhou, Ronggui Hu, Hiroaki Kiyokawa, Jun Yin, Bo Zhao

**Affiliations:** 1 Engineering Research Center of Cell and Therapeutic Antibody, Ministry of Education, School of Pharmacy, Shanghai Jiao Tong University, Shanghai 200240, China; pengkangli@sjtu.edu.cn; 2 Department of Chemistry, Center for Diagnostics and Therapeutics, Georgia State University, Atlanta, GA 30303, USA; rliu8@student.gsu.edu (R.L.); gjeong2@student.gsu.edu (G.H.J.); lzhou5@student.gsu.edu (L.Z.); 3 Center for Excellence in Molecular Cell Science, Chinese Academy of Sciences, Shanghai 100864, China; jiacaiwei@foxmail.com (C.J.); coryhu@sibcb.ac.cn (R.H.); 4 Department of Pathophysiology, School of Medicine, Jinan University, Guangzhou 510632, China; wangyiyang@jnu.edu.cn; 5 Department of Pharmacology, Northwestern University, Chicago, IL 60611, USA; kiyokawa@northwestern.edu

**Keywords:** ubiquitin, ubiquitination, O-GlcNAcylation, human papillomavirus, E6AP, E6, OGT, O-GlcNAc

## Abstract

Glycosyltransferase OGT catalyzes the conjugation of O-linked β-D-N-acetylglucosamine (O-GlcNAc) to Ser and Thr residues of the cellular proteins and regulates many key processes in the cell. Here, we report the identification of OGT as a ubiquitination target of HECT-type E3 ubiquitin (UB) ligase E6AP, whose overexpression in HEK293 cells would induce the degradation of OGT. We also found that the expression of E6AP in HeLa cells with the endogenous expression of the E6 protein of the human papillomavirus (HPV) would accelerate OGT degradation by the proteasome and suppress O-GlcNAc modification of OGT substrates in the cell. Overall, our study establishes a new mechanism of OGT regulation by the ubiquitin–proteasome system (UPS) that mediates the crosstalk between protein ubiquitination and O-GlcNAcylation pathways underlying diverse cellular processes.

## 1. Introduction

The dynamic modification of Ser and Thr residues of eukaryotic proteins by O-linked β-N-acetylglucosamine (O-GlcNAc) underpins diverse cellular processes such as gene regulation by the histone code, cell signaling by competing with protein phosphorylation, cell metabolism by sensing the nutrition status, or protein degradation by interacting with the ubiquitin–proteasome systems (UPS) [1,2,3]. O-GlcNAc modification (O-GlcNAcylation) of proteins is catalyzed by the O-GlcNAc transferase (OGT), which has multifaceted roles affecting UPS activity [4]. For example, OGT may suppress protein ubiquitination by adding O-GlcNAc to Ser or Thr residues within the degron motifs of the target proteins, so the degron sequence would not be phosphorylated at the same sites to mask their recognition by E3 ubiquitin (UB) ligases. Thus, O-GlcNAcylation of proteins would suppress their ubiquitination and degradation via the proteasome [5,6]. Furthermore, OGT may directly modify E3 UB ligase Nedd4 to induce its proteolytic processing and terminate its activity to catalyze protein ubiquitination in the cell [7,8]. OGT may also modify components of the proteasome and inhibit the degradation of ubiquitinated proteins [9]. On the other hand, various E3 UB ligases have been reported to ubiquitinate OGT to regulate its stability and activity in the cell. It was found that histone demethylase LSD2 could function as an E3 UB ligase through the binding between its zinc finger (ZnF) domain and the E2 UB-conjugating enzyme UbcH5c. This would enable UB transfer from LSD2 to OGT and induce the degradation of OGT [10]. Recently, Ring E3 XIAP was found to recognize OGT as a ubiquitination target and signal its degradation by the proteasome [11].

UB is a 76-residue protein that is transferred by E1-E2-E3 enzymatic cascades to the substrate proteins, and most often, UB is bounded to the substrate through the formation of isopeptide linkages between the C-terminal carboxylate of UB and the Lys residues of the substrate [12,13]. The first UB conjugated to the substrate can serve as the base for UB chain extension as the result of the repeated transfer of UB through the enzymatic cascades to the substrate proteins. UB chains of diverse linkages have been identified for protein modification, and the chains with K11 and K48 linkages are recognized by the proteasome for the degradation of the modified proteins [14,15]. To overcome the challenges in identifying the substrates of specific E3s that are responsible for recruiting substrates for UB modification, we developed a method known as “orthogonal UB transfer (OUT)” to enable the exclusive transfer of an engineered UB (xUB) through a defined E1-E2-E3 cascade to the substrate of a specific E3. Affinity-based purification of cellular proteins conjugated with xUB and their proteomic identification would enable us to profile the substrates of a specific E3 in the cell [16,17,18,19]. We previously assembled an OUT cascade of E6AP to profile its substrate specificity in HEK293 cells [20]. We found OGT was among the xUB-conjugated proteins purified from cells expressing the OUT cascade of E6AP, suggesting OGT could be recognized by E6AP as a substrate protein (Supplementary Table S1). E6 protein of the human papillomavirus (HPV) is known to stir the substrate specificity of E6AP in the host cells to facilitate virus survival and propagation [21]. Furthermore, E6 may function as an allosteric activator of E6AP to enhance its ubiquitination of the substrate proteins [22]. These results prompted us to assay whether OGT is regulated by E6AP through protein ubiquitination and if HPV E6 has a stimulatory effect on OGT ubiquitination catalyzed by E6AP. Here, we report the verification of OGT as an E6AP substrate and the enhancement of OGT ubiquitination by HPV E6 catalyzed by E6AP in vitro and in HeLa cells. We also found that co-expression of E6AP and E6 in the cell suppressed O-GlcNAcylation of OGT substrates by inducing the degradation of OGT via the proteasome. Our findings shed light on a new path for the crosstalk between protein O-GlcNAcylation and ubiquitination through the E6AP regulation of OGT stability in the cell and suggest HPV may affect protein O-GlcNAcylation in the host cells by stimulating the UB ligase activity of E6AP.

## 2. Results

### 2.1. E6 Stimulation of E6AP in Catalyzing OGT Ubiquitination In Vitro

**Figure 1:**
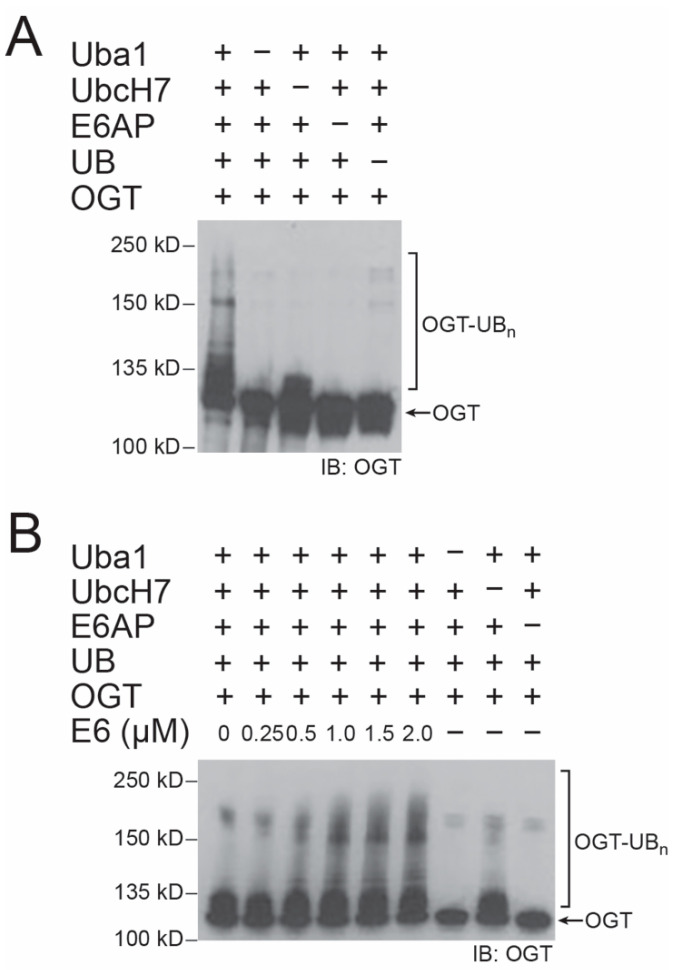
E6-mediated stimulation of OGT ubiquitination catalyzed by E6AP. (**A**) E6AP catalyzed OGT ubiquitination in reconstituted reactions in vitro. Control reactions were set up with Uba1, UbcH7, E6AP, or UB excluded from the reaction. Western blot of the reaction mixture was probed with an anti-OGT antibody. (**B**) HPV E6 enhanced the activity of E6AP in OGT ubiquitination. The ubiquitination reaction was set up with an increasing concentration of E6 in the range of 0–2 μM, and the level of OGT ubiquitination was probed with an anti-OGT antibody. The Western blot shown in (**B**) was exposed for a shorter time than that in (**A**) to reveal the difference of OGT ubiquitination in reactions with E6 added.

We expressed E6AP and OGT as 6× His tagged proteins from *E. coli* and set up ubiquitination reactions in vitro with and without E6 to verify OGT as an E6AP substrate. Without the addition of E6 to the reconstituted reaction, we found E6AP could ubiquitinate OGT with the formation of polyubiquitinated conjugates at the high molecular weight region (Figure 1A). The reaction was dependent on the presence of E1 (Uba1), E3 (E6AP), and UB. Mono-ubiquitination of OGT was observed when E2 (UbcH7) was excluded, suggesting there is low activity of UB loading from E1 to the HECT domain of E6AP that would further transfer UB to OGT. In the presence of E6, E6AP gained higher efficiency in the ubiquitination of OGT, and the enhancement of OGT ubiquitination was proportional to the amount of E6 added to the reconstituted system (Figure 1B). These results prove that E6AP would recognize OGT as a substrate protein for ubiquitination, and HPV E6 would have a stimulatory effect on OGT ubiquitination by E6AP.

We also co-expressed the UB transfer cascade of E6AP consisting of Uba1-UbcH7-E6AP and 6× His tagged OGT in *E. coli* cells to enable OGT ubiquitination in the cell. The OGT protein was then affinity purified by binding to Ni-NTA beads, and its ubiquitination level was measured by Western blotting probed with an anti-UB antibody. Strong ubiquitination of OGT was observed when the full E6AP cascade was expressed in the cell (Supplementary Figure S1A). In contrast, when E6AP was not expressed with OGT and the Uba1-UbcH7 pair in the cell, no OGT ubiquitination was observed, confirming the recognition of OGT by E6AP as a ubiquitination target. Ubiquitinated OGT bound to Ni-NTA beads was digested by trypsin, and the ubiquitination sites in the protein were identified by LC–MS based on the presence of diGly residue conjugated to the Lys residues of the OGT peptides (Supplementary Figure S1B). Fourteen OGT-derived peptides bearing the diGly modification were identified, and many of them match the ubiquitination sites of OGT documented in the PhosphoSite database (Supplementary Table S2) [23].

### 2.2. Regulation of OGT Stability in HEK293 Cells by E6AP

**Figure 2:**
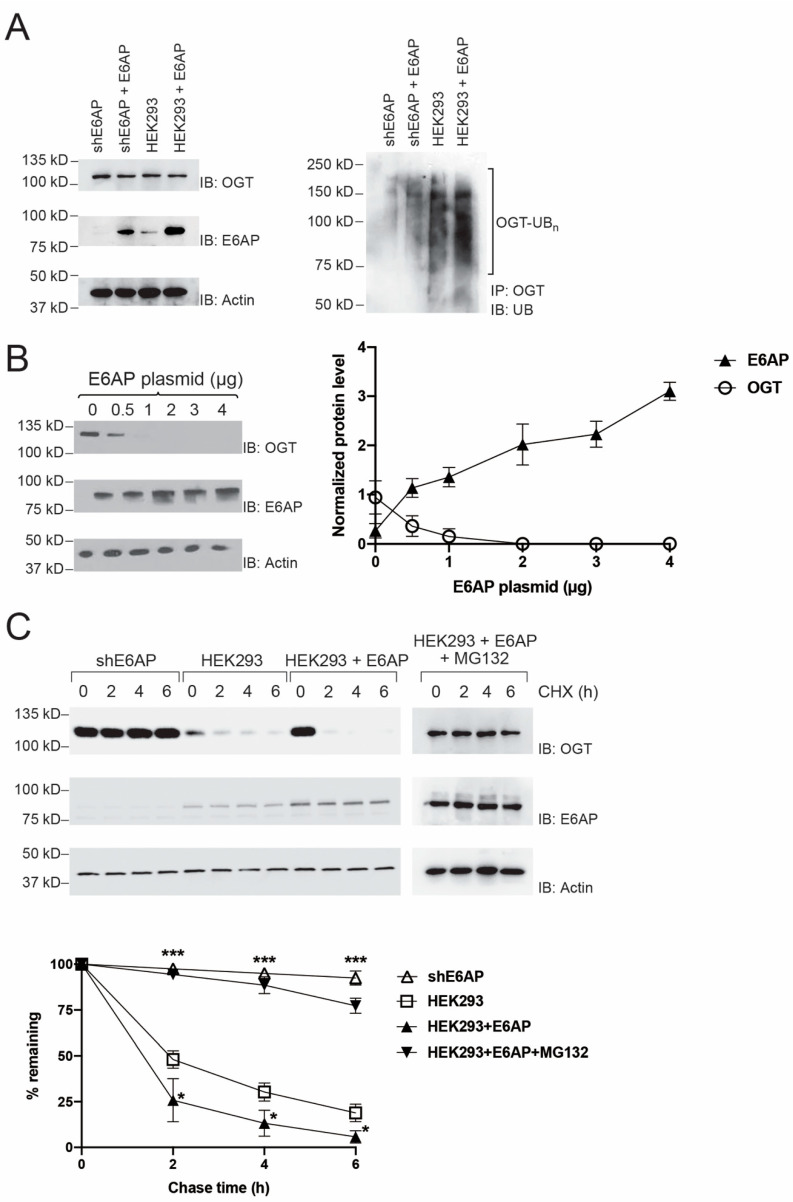
E6AP-catalyzed OGT ubiquitination and regulation of OGT stability in HEK293 cells. (**A**) Enhanced OGT ubiquitination with forced expression of E6AP in HEK293 cells. Left panels show inhibition of E6AP expression by anti-E6AP shRNA (shE6AP), and E6AP expression was restored by transfection of the plvx-E6AP plasmid into the shE6AP cells (shE6AP + E6AP). Additionally, HEK293 cells transfected with plvx-E6AP (HEK293 + E6AP) showed a higher level of E6AP expression than the blank cells (HEK293). The right panel shows the ubiquitination of OGT was enhanced with forced E6AP expression in the cells. The cells were treated with proteasome inhibitor MG132 for 4 h before the cell lysates were prepared for immunoprecipitation of OGT with an anti-OGT antibody. The precipitated samples were analyzed by SDS-PAGE and Western blotting with an anti-UB antibody. Ubiquitination of OGT was compared among HEK293 expressing shE6AP (shE6AP), HEK293 expressing both shE6AP and recombinant E6AP (shE6AP + E6AP), blank HEK293 cells (HEK293), and HEK293 expressing recombinant E6AP (HEK293 + E6AP). (**B**) E6AP decreased the steady-state level of OGT in HEK293 cells. Cells were transfected with different amounts of E6AP plasmid. Levels of OGT were assayed by immunoblotting of the cell lysates probed with an anti-OGT antibody. Quantitative analysis of OGT level in correlation with E6AP expression is plotted in the panel on the right. (**C**) E6AP-dependent degradation of OGT assayed by cycloheximide (CHX) chase. Cells were first treated with CHX to inhibit protein expression and harvested 0, 2, 4, and 6 h after incubation with CHX. OGT levels in the cell lysates were measured by immunoblotting with an anti-OGT antibody. The CHX chase assay was performed with shE6AP cells, blank HEK293 cells, HEK293 cells with E6AP overexpression (HEK293 + E6AP), and cells with E6AP overexpression and MG132 treatment (HEK293 + E6AP + MG132). Quantitative analysis of the OGT level in the cell was plotted in the panel underneath the blots. Data points show mean ± S.E. of three or more experiments. The vertical bars in (**B**,**C**) represent SEM from three independent experiments (n = 3). * *p* < 0.05 versus HEK293 control. *** *p* < 0.001 versus HEK293 control.

We then assayed OGT ubiquitination in HEK293 cells with suppressed or over-expressed E6AP to verify E6AP-catalyzed ubiquitination of OGT in the cell. We previously screened HEK293 cells with silenced E6AP expression with siRNA encoded by a shE6AP plasmid (shE6AP cells) [20]. We thus transfected plvx-E6AP plasmids into blank HEK293 and shE6AP cells for forced expression of the E3, immunoprecipitated OGT from the cell lysate with an anti-OGT antibody, and probed and compared the ubiquitination levels of OGT in cells with and without E6AP overexpression. We treated cells with 10 μM MG132 for 4 h before harvesting to inhibit proteasome activities and increase the accumulation of ubiquitinated species in the cell. We first assayed the expression of E6AP in cells with and without the transfection of plvx-E6AP. Indeed, the expression of E6AP was not detectable in shE6AP cells, but transfection of plvx-E6AP resulted in E6AP overexpression in shE6AP cells and HEK293 cells at a level higher than that of the endogenous E6AP (Figure 2A, left panel). We then performed immunoprecipitation of OGT followed by Western blotting probed with an anti-UB antibody to compare the amount of OGT ubiquitination in various cells. We found the ubiquitination of OGT was decreased in shE6AP cells comparing to the blank cells, yet overexpression of E6AP in the two types of cells would elevate the ubiquitination level of OGT (Figure 2A, right panel). These results suggest that E6AP would target OGT for ubiquitination in the HEK293 cells.

We further assayed E6AP regulation of OGT stability in the cells by transiently transfecting the HEK293 cells with increasing amounts of plvx-E6AP plasmids. We analyzed the steady-state levels of the OGT protein by Western blotting and found the decrease of OGT level was dose-dependent on the amount of E6AP expression in the cell (Figure 2B), indicating that E6AP would regulate the stability of OGT in HEK293 cells. We next measured the effect of E6AP expression on the half-life of OGT by carrying out cycloheximide (CHX) chase assay in shE6AP cells and HEK293 cells with and without the overexpression of E6AP. The cells were treated with 100 μg/mL CHX to inhibit protein synthesis and were collected at 0, 2, 4, and 6 h after the addition of CHX to analyze OGT levels by Western blotting. We found OGT level was stable in shE6AP cells with silenced E6AP expression, and in contrast, overexpression of E6AP would accelerate OGT degradation in the cells. Furthermore, the OGT level became stabilized in HEK293 cells with E6AP over-expression when the cells were treated with MG132 to inhibit proteasome activity (Figure 2C). These results collectively prove that E6AP catalyzes the ubiquitination of OGT and induces its degradation by the proteasome in HEK293 cells.

### 2.3. E6 stimulation of OGT Ubiquitination by E6AP

**Figure 3:**
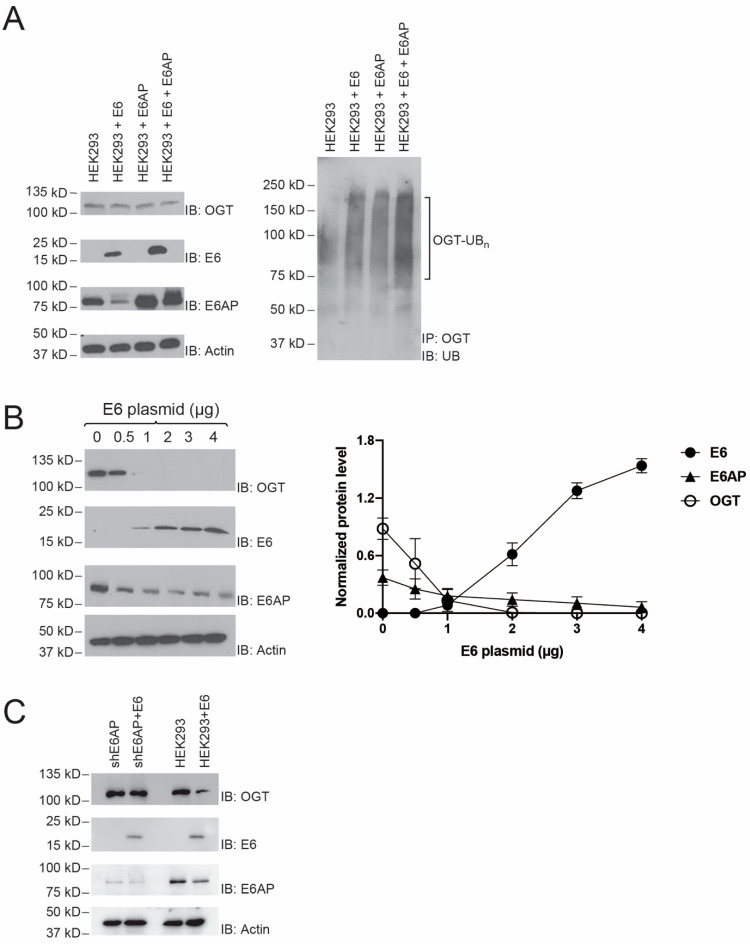
E6 enhanced OGT ubiquitination and degradation mediated by E6AP in HEK293 cells. (**A**) E6 enhanced OGT ubiquitination in the cells. HEK293 cells were transfected with plvx-E6AP or pLenti-E6 plasmids separately or together, and the expression of E6 and E6AP was confirmed by immunoblotting with an anti-E6 antibody or an anti-E6AP antibody (left panel). After 4 h treatment of MG132, ubiquitination of OGT was compared among blank HEK293 cells (HEK293), HEK293 cells expressing recombinant E6 (HEK293 + E6), HEK293 cells expressing recombinant E6AP (HEK293 + E6AP), and HEK293 cells expressing both recombinant E6AP and E6 (HEK293 + E6AP + E6) (right panel). OGT ubiquitination enhanced by E6 was confirmed by the elevated ubiquitination levels of OGT in cells with co-expression of E6AP and E6. (**B**) E6 decreased the steady-state level of OGT in HEK293 cells. Cells were transfected with different amounts of E6 plasmid. Levels of OGT were assayed by immunoblotting of the cell lysate probed with an anti-OGT antibody. Quantitative analysis of OGT level in correlation with the amount of E6 expression was plotted in the panel on the right. Data points show mean ± S.E. of three or more experiments. The vertical bars in (**B**) represent SEM from three independent experiments (n = 3). (**C**) The dependence on E6AP for regulating OGT level in the cells. shE6AP and HEK293 cells were transfected with or without E6 plasmid, and levels of OGT were measured by immunoblotting with an anti-OGT antibody.

Since the viral E6 protein may stimulate E6AP’s activity in UB transfer and redirect its ubiquitination targets [21,22], we assayed the effect of E6 on OGT ubiquitination catalyzed by E6AP in the cell. We transfected HEK293 cells with pLenti-E6 and plvx-E6AP plasmids to enable the expression of HPV E6 and E6AP together or alone (Figure 3A, left panel). We then treated the cells with proteasome inhibitor MG132 for 4 h and assayed the ubiquitination of OGT by immunoprecipitation and immunoblotting with an anti-OGT antibody and an anti-UB antibody, respectively. We found co-expression of E6 and E6AP could significantly enhance the ubiquitination of OGT comparing to the blank cells or cells with forced expression of either E6AP or E6 (Figure 3A, right panel). These results indicated that E6 could enhance OGT ubiquitination mediated by E6AP in HEK293 cells. To further measure the effect of E6 on the stability of OGT, different amounts of E6 plasmid were transfected into HEK293 cells, and the levels of OGT protein were determined by immunoblotting with an anti-OGT antibody. As the amount of E6 increased, the level of OGT decreased in a dose-dependent manner, indicating that E6 accelerated the degradation of OGT (Figure 3B). We also found that E6AP was unstable when there was a high level of E6 expression in the cell (Figure 3B, third panel from the top). Such results match with previous reports on E6 promoting the ubiquitination and degradation of E6AP in the cell [24,25]. Still, E6AP at the decreased level was able to pair with E6 and significantly destabilize OGT in the cell. To demonstrate E6’s dependence on E6AP to regulate OGT levels, we compared the steady-state levels of OGT in shE6AP cells and HEK293 cells with and without the expression of E6 (Figure 3C). The OGT level was stable in shE6AP cells with diminished E6AP expression and was not affected by the expression of E6. In contrast, the OGT level in HEK293 cells with endogenous E6AP expression was decreased by the co-expression of E6. These results suggest E6 regulates OGT levels in the cell through E6AP-mediated ubiquitination.

### 2.4. Effect of E6AP Activity on Protein O-GlcNAylation in HeLa Cells

**Figure 4:**
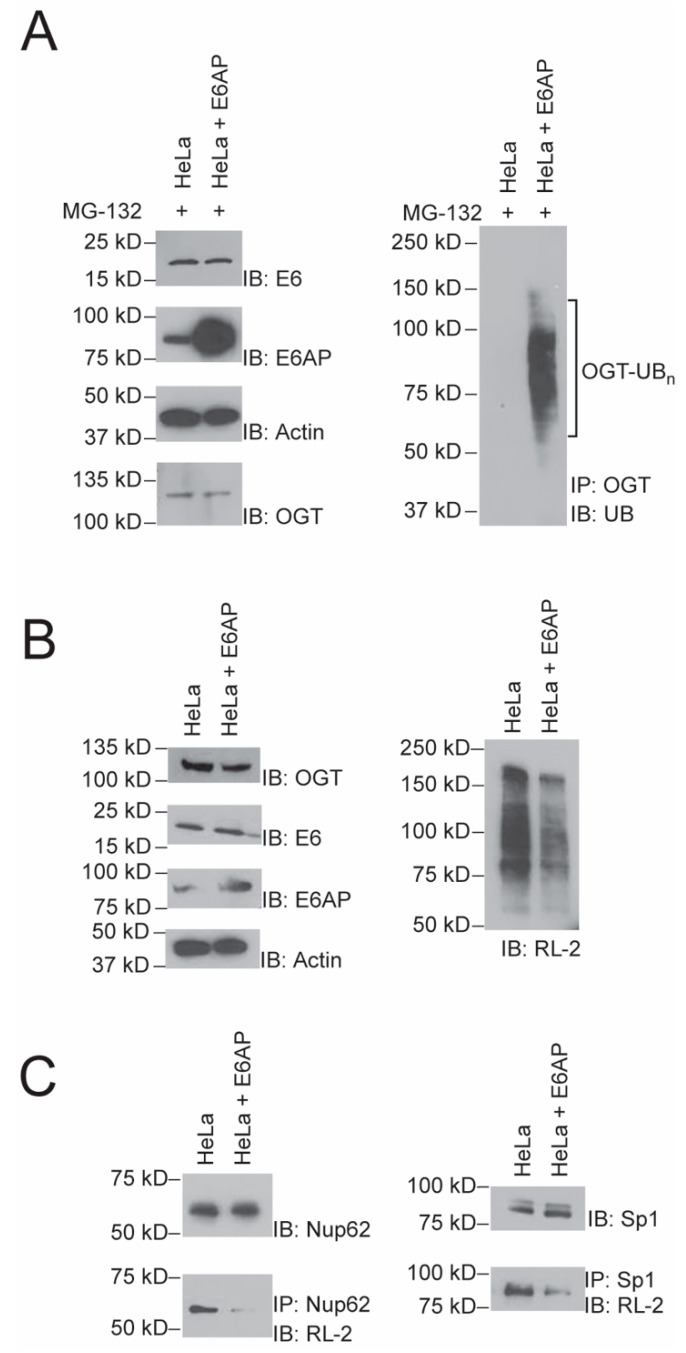
E6AP regulated protein O-GlcNAcylation in HeLa cells by inducing the degradation of OGT. (**A**) Overexpression of E6AP in the presence of proteasome inhibitor MG132 in Hela cells was confirmed by immunoblotting with an anti-E6AP antibody (left panels). Ubiquitination of OGT in the presence of MG132 in HeLa cells was assayed by immunoprecipitation with an anti-OGT antibody and immunoblotting with an anti-UB antibody (right panel). Ubiquitination of OGT was compared between blank HeLa cells (HeLa) and HeLa cells expressing recombinant E6AP (Hela + E6AP). (**B**) Levels of OGT and protein O-GlcNAylation compared between HeLa and HeLa + E6AP cells. OGT levels were probed in two types of cells on the Western blot with an anti-OGT antibody (left panels). The level of O-GlcNAc modification of cellular proteins was detected with an anti-O-GlcNAc antibody (RL-2) (right panel). (**C**) E6AP expression negatively regulated the O-GlcNAcylation of two known OGT substrates, Nup62 and Sp1. Cell lysates of HeLa and HeLa + E6AP cells were immunoprecipitated with substrate-specific antibodies (anti-Nup62 or anti-Sp1) and immunoblotted with the RL-2 antibody to detect the levels of O-GlcNAc-modified proteins. In (**B**,**C**), cells were not treated with MG132 to allow the degradation of OGT induced by ubiquitination catalyzed by the E6AP–E6 pair.

After the verification of the E6–E6AP mediated ubiquitination and degradation of OGT, we assayed if the enhancement of E6AP activity by E6 in the cell would affect protein O-GlcNAylation. Since HeLa cells stably express E6 originated from HPV infection [26], we transiently transfected plvx-E6AP into Hela cells and verified the overexpression of E6AP comparing to the blank cell (Figure 4A, left panel). When the cells were treated with MG132 to inhibit proteasome activity, there was more accumulation of polyubiquitinated OGT in cells with E6AP expression, suggesting the E6–E6AP pair would stimulate OGT ubiquitination in the cells (Figure 4A, right panel). In cells overexpressing E6AP without proteosome inhibition, we found there was a decreased level of OGT, and correspondingly, the level of protein O-GlcNAcylation was significantly decreased upon probing the Western blot of the cell lysates with an anti-O-GlcNAc antibody (RL-2) (Figure 4B)**.** Finally, we assayed if the induced degradation of OGT by E6–E6AP would affect the O-GlcNAc modification of specific OGT substrates. We chose a nuclear complex component Nup62 and a transcription factor Sp1 with known O-GlcNAcylaion sites as model substrates of OGT [27,28,29,30,31]. The two proteins were immunoprecipitated from the lysates of HeLa cells with or without E6AP over-expression. As expected, we found that Nup62 and Sp1 showed less O-GlcNAc modification in HeLa cells with forced expression of E6AP (Figure 4C), suggesting that the E6–E6AP pair could induce ubiquitination and degradation of OGT in Hela cells, therefore regulating the level of protein O-GlcNAcylation in the cell.

## 3. Discussion

Similar to phosphorylation, O-GlcNAc modification can dynamically change in response to a myriad of stimuli and cellular environment. Both O-GlcNAcylation and phosphorylation act on the Ser/Thr sites, so on many proteins, the two types of modification would compete and carry counterbalancing cellular signals [32]. There are only two enzymes that regulate O-GlcNAc modification in the cell, namely, OGT catalyzing the addition of O-GlcNAc and β-N-acetylglucosaminidase O-GlcNAcase (OGA) catalyzing the removal of O-GlcNAc [33]. Perturbations in the metabolism of O-GlcNAc and alterations of O-GlcNAcylated proteins are associated with Alzheimer’s disease, diabetes [34], and cancer [35]. A number of E3 UB ligases have been reported to interact with OGT and regulate its activity in the cell. In addition to histone methyltransferase, LSD2 and Ring E3 XIAP that have been shown to ubiquitinate OGT in reconstituted ubiquitination assays in vitro and in the cell [10,11]. β-TrCP, a substrate adaptor of the Skp1–Cullin–F-box-protein (SCF) E3, was found to destabilize OGT in the cell, although the ubiquitination of OGT catalyzed by the SCF complex bridged by β-TrCP is yet to be confirmed [36]. Hsp90, a chaperone protein associated with E3 UB ligase CHIP, was found to interact with OGT and regulate its expression level in the cell. But CHIP was ruled out to ubiquitinate OGT and induce its degradation [37]. It was also found OGT would interact with E3 ligase EEL-1 in the neuronal cells of *C. elegans*, yet it is not known if EEL-1, an ortholog of human E3 Huwe1, would target OGT for ubiquitination [38]. In this report, we verified the activity of E6AP in ubiquitinating OGT and regulating its stability in the cell. We also found HPV E6 could significantly enhance the UB ligase activity of E6AP toward OGT and suppress the cellular O-GlcNAcylation level by coercing OGT for degradation.

Previously, HPV E6 was found to up-regulate the expression of OGT by elevating the transcription of the OGT gene in cervical cancer tissues. The high OGT activity in cancer cells would enhance O-GlcNAcylation of the c-myc oncoprotein and stabilize c-myc to promote cancer cell transformation and metastasis [39]. It was also found OGT expression would enhance the expression levels of HPV E6 and E7 proteins in cervical cancer cells [40], so it seems E6 and OGT are in a feedforward cycle to enhance each other’s activity. Our finding of enhanced OGT ubiquitination and destabilization stimulated by E6 suggests that E6 may take a different role in suppressing OGT activity by pairing with E6AP. E6-stimulated OGT ubiquitination and degradation may help to counteract the antiviral activity of OGT that has been identified as a part of the host defense mechanism against viral infection by hepatitis B virus (HBV), Kaposi’s sarcoma-associated herpesvirus (KSHV), respiratory syncytial virus (RSV), and human immunodeficiency virus (HIV-1) [40,41,42,43,44,45]. OGT has been found to enhance the autophagy activity of the host cells through which the HBV viral particles would be degraded, and it can also down-regulate gene expression from the HIV genome inside the host cells [41,42,43,44]. It would be of interest to further test if OGT ubiquitination mediated by the E6–E6AP pair would enhance HPV replication in the host cells by suppressing the antiviral activity of OGT. In addition to E6AP, E6 may pair with other E3s such as EDD1 to ubiquitinate cellular targets [46,47]. Therefore, future studies may identify other E3s regulating OGT in the cell under the influence of HPV E6.

## 4. Materials and Methods

### 4.1. Plasmids

The following expression plasmids were used: pcDNA5FRT/FLAG-OGT (29760) and pLenti6.3 HA/FLAG V42L HPV 16E6 (37445) were from Addgene (Cambridge, MA, USA); plvx-IRES-mcherry empty vector was a kind gift from Feng Qian’s lab of Shanghai Jiao Tong University; plvx-E6AP-IRES-mcherry was generated by insertion of full-length E6AP gene between the SpeI and BamHI restriction sites of the plvx-IRES-mcherry vector.

### 4.2. Antibodies

The following antibodies were purchased from Santa Cruz Biotechnology (Dallas, TX, USA): anti-OGT (sc-74546), anti-E6AP (sc-166689), anti-hemagglutinin (HA) (sc-7392), anti-actin (sc-8432), anti-UB (sc-8017), and mouse anti-rabbit IgG-HRP (sc-2357). Goat anti-mouse IgG secondary antibody (31438) was from Thermo Fisher Scientific (Waltham, MA, USA). Anti-FLAG (M2) antibody (F3165) was from Sigma-Aldrich (Burlington, MA, USA). Anti-Nup62 antibody (NBP1-31381) was from Novus Biologicals (Centennial, CO, USA). Anti-Sp1 antibody (07-645) was from EMD Millipore (Burlington, MA, USA). Anti-O-GlcNAc RL2 antibody (2739) was from Abcam (Cambridge, MA, USA). E6 protein of human papillomavirus type 16 was from Boston Biochem (Cambridge, MA, USA).

### 4.3. Expression and Purification of Recombinant Proteins

6× His tagged proteins were expressed in *E.coli* BL21 cells. After induction with 1 mM IPTG, the cells were collected by centrifugation, and the pellets were lysed and resuspended in lysis buffer (50 mM Tris-Base, 500 mM NaCl, 5 mM imidazole, pH 8.0), followed by sonication and centrifugation to remove cell debris. Bacterial cell lysates were loaded onto a Ni-NTA column (Qiagen, Hilden, Germany) and rocked gently at 4 °C for 2 h. The bounded proteins were washed twice and eluted with 250 mM imidazole. Eluted proteins were dialyzed and analyzed by SDS-PAGE with Coomassie Brilliant Blue staining to confirm the size of expressed proteins.

### 4.4. In Vitro Ubiquitination of OGT

To assay the ubiquitination of OGT, 1 μM OGT was incubated with 1 μM Uba1, 5 μM UbcH7, 5 μM E6AP, 0–2 μM E6, 50 μM UB in TBS (137 mM NaCl, 2.7 mM KCl, and 24.8 mM Tris-Base, pH 7.4) supplemented with 10 mM MgCl_2_ and 1.5 mM ATP. The reactions were conducted in a total volume of 50 μL for 2 h at 37 °C. Total reaction mixtures were analyzed by 4–15% SDS-PAGE, followed by Western blotting probed with an anti-OGT antibody.

### 4.5. Identification of the Ubiquitination Sites of OGT

The genes of HA-tagged UB, Uba1, UbcH7 and E6AP were cloned into pACYCDuet-1 vector to generate pACYC-UB-Uba1-UbcH7-E6AP. Each gene was inserted into the multiple cloning sites to form the polycistron to express each protein under the control of a lac operator and a T7 promoter. pACYC-UB-Uba1-UbcH7 was also constructed as a control. The OGT gene was cloned into pET-28a-6× His vector to generate pET-His-OGT. BL21 (DE3) pLysS competent cells (Invitrogen, Carlsbad, CA, USA) were co-transformed with pET-His-OGT and pACYC-UB-Uba1-UbcH7-E6AP or pACYC-UB-Uba1-UbcH7. Single colonies of the transformed cells were grown on lysogeny broth (LB) plates supplemented with ampicillin (100 μg/mL) and kanamycin (50 μg/mL). Then, 1 L LB medium was inoculated with the overnight culture of the colonies and allowed to grow at 37 °C to an OD 0.8 following the addition of IPTG (0.25 mM). The culture was induced to express the proteins at 16 °C for 18 h, and the cells were harvested and resuspended in urea buffer (8 M urea, 300 mM NaCl, 50 mM Na_2_HPO_4_, 50 mM Tris-Base, 0.5% NP40, pH 8.0) and lysed by sonication. The suspension was centrifuged at 12,000 r.p.m. to remove the cell debris and purified by binding to 1 mL Ni-NTA agarose beads. Proteins bound to the Ni-NTA beads were eluted and analyzed by SDS-PAGE followed by immunoblotting with an anti-His antibody to detect the expression of OGT and an anti-HA antibody to detect the ubiquitination of OGT. The crude cell lysate was analyzed by SDS-PAGE and immunoblotting with an anti-E6AP antibody to detect the expression of E6AP.

For mapping ubiquitination sites on OGT by MS, the cell lysate purified by Ni-NTA agarose beads (first immunoprecipitation) was dialyzed overnight at 4 °C in RIPA buffer (150 mM NaCl, 50 mM Tris-Base, 1% NP40, 0.1% SDS, 5 mM EDTA, 0.5% sodium deoxycholate, pH 7.5). After washing 5 times with urea buffer, the proteins on the beads were eluted with elution buffer (150 mM NaCl, 50 mM Tris-Base, 1% NP40, 0.1% SDS, 5 mM EDTA, 0.5% sodium deoxycholate, 1 M imidazole, pH 8). The eluent was dialyzed overnight at 4 °C in RIPA buffer, then bound with anti-HA beads for 6 h for the second immunoprecipitation. The anti-HA beads were washed 5 times with RIPA buffer, then reduced by tris (2-carboxyethyl) phosphine (TCEP) and alkylated by N-ethylmaleimide (NEM), following the digestion by trypsin. The digested peptides were separated by the EASY-nLC 1000 system (Thermo Fisher, Waltham, MA, USA) and analyzed by the Q Exactive mass spectrometer (Thermo Fisher, Waltham, MA, USA). Protein and ubiquitination analyses were performed with Thermo Proteome Discoverer 2.1 (Thermo Fisher), and the peptide fragments were searched against Uniprot Human database (http://www.uniprot.org/, accessed on 2 February 2021). The ubiquitination sites were identified based on the increase of the molecular weight on lysine (K) residues (+114.04) due to conjugation with diGly.

### 4.6. Cell Culture and Transfection

HEK293 cells and HeLa cells were cultured in high-glucose Dulbecco’s modified Eagles medium (DMEM) from Life Technologies (Carlsbad, CA, USA) with 10% fetal bovine serum (FBS). Transient transfection of cells was performed with Dharmacon transfection reagent (Dharmacon T-2006-01, Lafayette, CO, USA), according to the manufacturer’s recommendations.

### 4.7. Coimmunoprecipitation to Confirm OGT Ubiquitination in HEK 293 Cells

HEK293 cells (60–70% confluent monolayer in a 75-cm^2^ cell culture flask) were transfected with 8 μg plvx-mcherry E6AP or 8 μg plvx-mcherry empty vector with 2 μg pcDNA-OGT to increase OGT expression. Forty-eight hours after transfection, shE6AP, shE6AP + E6AP, HEK293, and HEK293 + E6AP cells were treated with 10 μM MG132 for 4 h. Cells were washed twice with cold PBS and lysed in RIPA buffer. After centrifugation at 13,000 r.p.m. for 30 min at 4 °C, the supernatant was precleared by adding 1 μg of the appropriate control IgG. Then, 20 μL Protein A/G PLUS agarose (sc-2003) was also added to the supernatant and incubated for 30 min at 4 °C, followed by centrifugation at 350× *g* for 5 min at 4 °C. The concentration of cleared cell lysate was measured. An amount of 2 mg of the total cell lysate was transferred to a new tube, and 4 μg OGT antibody was added to the tube. The incubation was continued for 1 h at 4 °C. After incubation, 40 μL resuspended protein A/G PLUS agarose was added to the tube, and the tube was put on a rocking platform for incubation overnight at 4 °C. The next day, the beads were washed 4 times with 1 mL cold PBS each time. After the final wash, the beads were resuspended in 30 μL PBS together with 6 μL 6 × loading dye, and the samples were boiled for 10 min. After brief centrifugation, the supernatant was analyzed by 4–15% SDS-PAGE, and the Western blot of the gel was probed with an anti-UB antibody to measure the ubiquitination level of OGT.

### 4.8. OGT Degradation Assays

For experiments to measure the effect of E6AP on OGT degradation in HEK293 cells, cells were transiently transfected with 0.5, 1, 2, 3, and 4 μg plvx-E6AP. Cells were harvested in RIPA buffer 48 h after transfection, and the level of OGT protein was assayed by immunoblotting with an anti-OGT antibody. For assaying the steady-state levels of OGT with E6 expression, HEK293 cells were transfected with different amounts of pLenti-E6 plasmid, cultured for 48 h. The cells were then harvested, and the levels of OGT protein were analyzed by Western blotting probed with an anti-OGT antibody. For assaying the dependence of OGT stability on E6AP, shE6AP and HEK293 cells were transfected with the pLenti-E6 plasmid, and the OGT levels in the cells 48 h after transfection were measured by Western blotting probed with an anti-OGT antibody. The OGT levels in shE6AP and HEK293 cells with E6 expression were compared to the corresponding cells without the transfection of the pLenti-E6 plasmid to reveal the difference.

### 4.9. Coimmunoprecipitation to Confirm E6AP Regulation of Protein O-GlcNAcylation

HeLa cells (60–70% confluent monolayer in a 75-cm^2^ cell culture flask) were transiently transfected with 8 μg plvx-mcherry E6AP or 8 μg plvx-mcherry empty vector as a control. At 48 h post-transfection, cells were harvested in RIPA buffer. The cell lysate was immunoprecipitated with an anti-Nup62 or anti-Sp1 antibody and immunoblotted with an anti-O-GlcNAc RL2 antibody. The total cell lysates were resolved by SDS-PAGE, and Western blots of the gels were probed with an anti-OGT or RL-2 antibody to detect OGT or O-GlcNAcylated proteins.

### 4.10. Statistical Analysis

All statistical analyses were performed using Graphpad Prism (Graphpad prism 9.0.0 software, San Diego, CA, USA). All quantitative data were presented as mean ± SEM. Differences between the two groups were assessed by unpaired Student’s *t*-test. *p* < 0.05 was considered statistically significant.

## Data Availability

All data in this study are included in the main manuscript and in the supplementary materials.

## Supplementary Materials

The following are available online at https://www.mdpi.com/article/10.3390/ijms221910286/s1.

## Abbreviations

CHX	cycloheximide
HPV	human papillomavirus
OGT	O-linked N-acetyl glucosamine transferase
O-GlcNAcylation	O-GlcNAc modification
OUT	orthogonal ubiquitin transfer
RL-2	anti-O-GlcNAc antibody
UB	ubiquitin
UPS	ubiquitin–proteasome system
