# Supplementary material for: Regulation of O-Linked N-Acetyl Glucosamine Transferase (OGT) through E6 Stimulation of the Ubiquitin Ligase Activity of E6AP
Source: Int J Mol Sci. 2021 Sep 24;22(19):10286. doi: 10.3390/ijms221910286 (PMC8508608; doi:10.3390/ijms221910286)
Supplement: Supplementary file 1 [file ijms-22-10286-s001.zip › Supplementary Material.pdf]

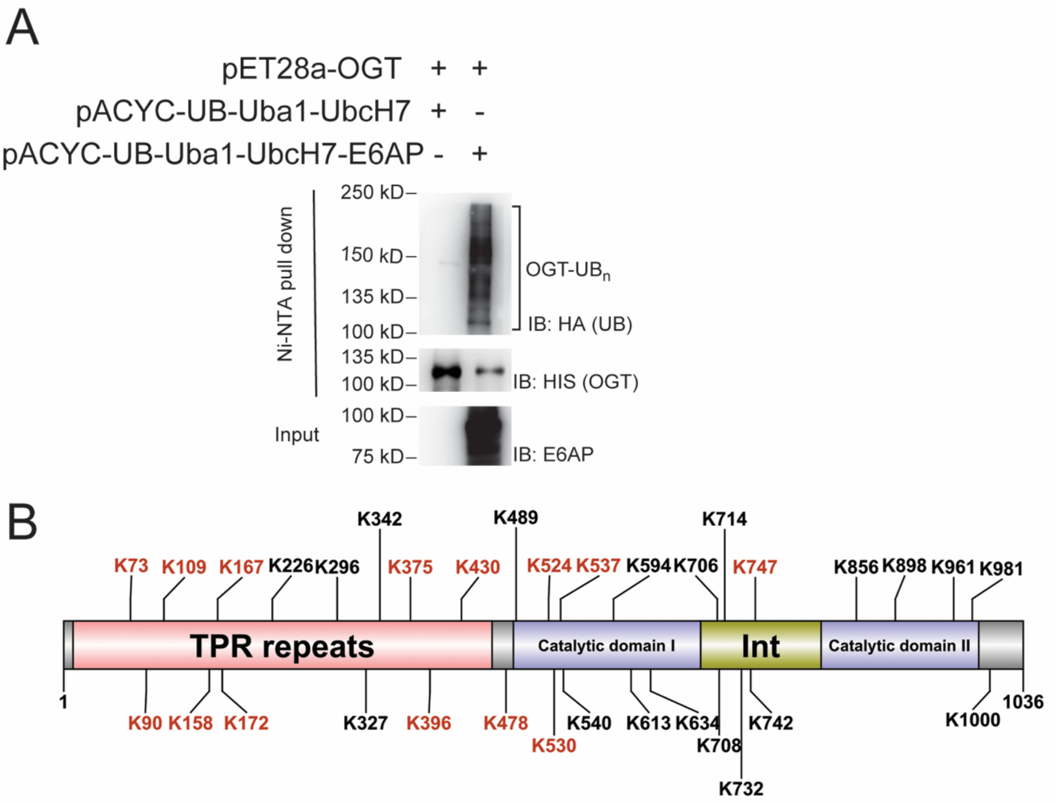

Figure S1. Identification of the ubiquitination sites of OGT with the coexpression of OGT with E6AP ubiquitination cascade in E coli cells. (A) BL21 *E.coli* cells were co-transformed with pACYC-UB-Uba1-UbcH7-E6AP and pET-28a-His-OGT to express 6×His tagged OGT with the E6AP cascade in the cell. OGT was pulled down by Ni-NTA agarose beads and subjected to immunoblotting analysis by an anti-HA antibody that would detect the HA tag fused to UB. As a control, OGT was coexpressed with Uba1 and UbcH7 but without E6AP in *E coli* cells for measuring the ubiquitination level of OGT. (B) Schematic illustration of the 14 lysine residues (K) in OGT identified as the sites of E6AP catalyzed ubiquitination reaction. Ubiquitination sites identified in this study are shown in red. Ubiquitination sites not found in this study but documented in the PhosphoSite database are shown in black.

Table S1. Proteomics data for identifying OGT as an E6AP substrate by Orthogonal Ubiquitin Transfer (OUT).See separate Excel file.

Table S2. E6AP-targeted protein ubiquitination sites in OGT identified by LC-MS.

**Sites on OGT**	**Peptide sequences with diGly modification identified by LC-MS**	**Sites documented in PhosphoSites.org**
73	RSAHFSTLAIKQNPLLA	YES
90	KQNPLLAEAYSNLGNVYKERG	YES
109	LRLKPDFIDGYINLAAALVAAGDM	NO
158	RSDLGNLLKALGRL	YES
167	RLEEAKACYLKA	YES
172	YLKAIETQPNFAVA	NO
375	KLQEALMHYKEAIRI	YES
396	ISPTFADAYSNMGNTLKEMQD	NO
430	RAIQINPAFADAHSNLASIHKDSGNIPEAIASYRT	YES
478	KKLVSIVADQLEKN	YES

524	RHGNLCLDKINVLHKPPYEHPKDLKL	YES
530	KINVLHKPPYEHPKD	YES
537	KINVLHKPPYEHPKDLKL	YES
747	MKCPDGGDNAD	NO
